# Intrinsic, extrinsic and interaction theories on falls and aging-in-place: A C-T-E scoping review

**DOI:** 10.1093/geroni/igab046.2358

**Published:** 2021-12-17

**Authors:** Bonnie Albright

**Affiliations:** University of Massachusetts - Boston, Boston, Massachusetts, United States

## Abstract

As aging in place increases in popularity, it is important to understand potential negative outcomes related to the trend. For this presentation, the conceptual-theoretical-empirical (C-T-E) scoping review technique was used to organize research on in-home falls of community-dwelling older adults. Research and theory were included from the fields of social gerontology, disability, policy, social justice, medicine, rehabilitation, and housing. While research from these multiple fields overlaps, an overarching conceptual framework for organizing this literature was found to categorize the theories into three main conceptual areas. The three conceptual areas are: intrinsic (related to the person only), extrinsic (related to external factors only), and interaction between intrinsic and extrinsic (related to the interaction between the person and external factors). This conceptual framework shares similarities with work by others in use of the terms intrinsic and extrinsic, and it draws on the larger influence of Bronfenbrenner's socio-ecological model. However, this review extends previous work by providing a framework for organizing the contributions to falls research across multiple disciplines.

